# Buruli Ulcer Recurrence, Benin

**DOI:** 10.3201/eid1104.041000

**Published:** 2005-04

**Authors:** Martine Debacker, Julia Aguiar, Christian Steunou, Claude Zinsou, Wayne M. Meyers, Françoise Portaels

**Affiliations:** * Institute of Tropical Medicine, Antwerp, Belgium;; † Centre Sanitaire et Nutritionnel Gbemoten, Zagnanado, Benin;; ‡ Armed Forces Institute of Pathology, Washington, DC, USA

**Keywords:** Mycobacterium ulcerans, Benin, follow-up, recurrence rate, Buruli ulcer, research

## Abstract

Buruli ulcer is a recognized public health problem in West Africa. In Benin, from 1989 to 2001, the Centre Sanitaire et Nutritionnel Gbemoten (CSNG) treated >2,500 Buruli ulcer patients. From March 2000 to February 2001, field trips were conducted in the Zou and Atlantique regions. The choice of the 2 regions was based on the distance from CSNG and on villages with the highest number of patients treated at CSNG. A total of 66 (44.0%) of 150 former patients treated at CSNG were located in the visited villages. The recurrence rate of CSNG-treated patients after a follow-up period of up to 7 years was low (6.1%, 95% confidence interval [CI] 2.0–15.6). We attribute this low rate to the high quality of Buruli ulcer treatment at an accessible regional center (CSNG). The World Health Organization definition of a Buruli ulcer recurrent case should be revised to include a follow-up period >1 year.

## Abstract 2

Access to high-quality treatment prevents Buruli ulcer recurrence.

*Mycobacterium ulcerans* disease, also called Buruli ulcer, is a recognized public health problem in many countries, especially in West Africa, where prevalence has been increasing in recent years (1,2). Buruli ulcer–endemic foci are regularly associated with stagnant bodies of water (ponds, backwaters, and swamps). The disease takes various clinical forms, including ulcers, nodules, plaques, and edematous indurations. Surgical excision followed by skin grafting is the recommended treatment (3). Recent studies, however, suggest that an antimicrobial regimen of rifampicin (rifampin) plus streptomycin may be effective against early forms of Buruli ulcer (4). Follow-up data on the rate of recurrence in hospital-treated Buruli ulcer patients are rarely reported. In a study designed to assess the effectiveness of excising preulcerative Buruli lesions in field situations in Ghana, Amofah et al. found a local recurrence rate of 16% at the same site within a year of follow-up (5). Two more patients had a recurrence at a different site, for a total recurrence rate of 20%. In villages in Ghana, Teelken et al. compiled a group of 78 patients who had been treated in 2 different hospitals: 35% were not healed when followed up 3 years later. For 1 hospital, the rate of those not healed was 18%, and in the other, 47%. In their study, however, investigators were not able to differentiate between ulcers that had never healed and those that healed and then recurred (6).

Our village follow-up was organized and carried out by the Centre Sanitaire et Nutritionnel Gbemoten (CSNG), a rural health center in southern Benin that began surgically treating patients in 1989 (7). This study reports the recurrence rate of CSNG-treated patients after up to 7 years of follow-up.

CSNG, in the district of Zagnanado, is one of several reference centers for the treatment of Buruli ulcer in Benin. Situated in the Zou region, 1 of 4 Buruli ulcer–endemic regions of southern Benin, this center receives Buruli ulcer patients from Benin and neighboring countries. From 1989 to 2001, CSNG treated 2,564 Buruli ulcer patients (2): 1,801 were from Zou, 170 were from Atlantique, and 515 were from other regions of southern Benin. The origin of 78 patients was not recorded. From March 2000 to February 2001, field trips in villages of the Atlantique and Zou regions were organized to collect data on rates of recurrence of disease and inform the population of the Buruli ulcer public health problem. CNSG could not collect data before 2000 because of a lack of transportation. In a previous study, the origin and number of patients coming to CNSG for treatment were described (2). We showed that most Buruli ulcer patients admitted to CSNG from 1997 to 2001 were from Zou, and <10% were from the adjacent Atlantique region (2). For villagers living at a distance from CSNG, transportation and treatment costs represent a considerable concern, but the greatest expenditure for patients is the cost of living at CSNG, especially the cost of food (8).

## Methods

From March 2000 to February 2001, 22 field trips were conducted in Zou and Atlantique. Eleven field trips were required to inform the local authorities about the objectives of the study, notify the population, and inform them of the time and date of the follow-up visit 15 days later. Detecting Buruli ulcer patients required 11 additional field trips.

Villages that were included in the study had to be in a region close to CSNG (Zou), where villagers were aware of the availability of a Buruli ulcer treatment center, or in a region far from CSNG (Atlantique), where distance to the center was a substantial problem. In each region, a list of patients from each region was compiled, and the districts and villages with the highest number of patients treated at CSNG were selected.

The field team included a staff physician of the CSNG, a microbiologist, and a driver. Publicity campaigns on Buruli ulcer were presented by CSNG in some villages in these regions. Before field trips, we compiled a list of Buruli ulcer patients from villages where publicity campaigns were organized. These patients were treated at CSNG at some point from 1989 to 2001. They were examined and interviewed with the cooperation, when necessary, of other persons, such as former patients treated at CSNG, teachers, parents, or for children, someone designated as representative of specific patients. These key persons helped us locate patients on our lists and sometimes find new patients and patients treated by traditional methods. All adolescent and adult participants were personally interviewed. For children, a competent adult who knew the study patient well enough to supply the requested information was interviewed. All patients, or parents of children included in the study, provided oral consent. Photographs were taken of some patients.

Posttreatment histories were taken in the language of each patient, either directly by the interviewer or through an interpreter, when necessary. Recurrences of Buruli ulcer symptoms were noted, specifically with respect to types and sites of new lesions and time between hospital discharge and follow-up. All patients with active Buruli ulcer were referred to CSNG.

Clinical diagnosis of 49 cases was confirmed by laboratory tests according to World Health Organization (WHO) guidelines (9). The remaining 17 cases were diagnosed clinically; all were typical Buruli ulcer and did not present reasonable differential diagnostic problems.

## Results

Table 1 shows the total number of villages (173) in the 3 Buruli ulcer–endemic districts (28 in Ouinhi, 74 in Zogbodomey, and 71 in Zè). From 1989 to 2001, a total of 70 villages (40%) were Buruli ulcer–endemic in the Zou and Atlantique regions. Two districts from the Zou region, Ouinhi and Zogbodomey, had 21 and 24 Buruli ulcer–endemic villages, respectively, and in the Atlantique region, 1 district (Zè) had 25 Buruli ulcer–endemic villages. Of 70 Buruli ulcer–endemic villages, 24 (34.3%) could be visited from March 2000 to February 2001, 13 (28.9%) in Zou and 11 (44.0%) in Atlantique.

**Table 1:** Visited villages and patients retrieved in 2 districts of the Zou region and 1 district of the Atlantique region

Villages*	Zou		Atlantique	
	Ouinhi	Zogbodomey	Zè	Total
Total villages	28	74	71	173
Total BU-endemic villages, 1989–2001	21	24	25	70
BU-endemic villages visited (%)	7 (33.3)	6 (25.0)	11 (44.0)	24 (34.3)
Total BU patients treated at CSNG, 1989–2001	419	170	118	707
BU patients previously treated at CSNG, 1989–2001, in visited villages	74	39	37	150
BU patients followed-up in visited villages (% retrieved)	24 (32.4)	17 (43.5)	25 (67.6)	66 (44.0)

*BU, Buruli ulcer; CSNG, Centre Sanitaire et Nutritionnel Gbemoten.

A total of 707 patients treated at CSNG originated in these districts: 419 from Ouinhi, 170 from Zogbodomey, and 118 from Zè. A total of 150 patients came from visited villages: 74 from Ouinhi, 39 from Zogbodomey, and 37 from Zè. A total of 66 (44.0%) of 150 Buruli ulcer patients formerly treated at CSNG were located, 41 (36.3 %) from Zou and 25 (67.6%) from Atlantique. The difference between the percentage of patients retrieved in Zou and Atlantique is significant (p < 0.001). We incidentally found 11 patients treated or under treatment by traditional healers and 28 new patients (data not shown).

The follow-up period (time between discharge and the follow-up visit) is indicated in Table 2. A total of 45 patients (73.8%) had at least 12 months of follow-up time (median 34 months), 12 patients (19.7%) had 6–11 months of follow-up time (median 9 months), and 4 patients (6.6%) had 2–5 months of follow-up time (median 4 months). The shortest period between hospital discharge and follow-up visit was 2 months, and the longest period was 7 years. For 5 patients, the exact date of discharge from the hospital was not recorded. All 5 patients had bone lesions, had been hospitalized several times, and had been discharged >1 year before the visit.

**Table 2:** Follow-up period for Buruli ulcer patients in villages of the Atlantique and Zou regions, Benin

Period	No. patients (%)	Median follow-up period (Q1–Q3) (mo)*
2–5 mo	4 (6.6)	4 (2.5–4.0)
6–11 mo	12 (19.7)	9 (7.25–10.75)
12 mo–7 y	45 (73.8)	34 (19.0–44.5)
Total	61	
>1 year†	5	

*Q1, first quartile; Q3, third quartile. †Exact date of discharge unknown.

Of the 66 patients treated at CSNG, 4 (6.1%, 95% confidence interval [CI] 2.0–15.6) had a new Buruli lesion. One patient (4.0%) of 25 came from Atlantique and 3 (7.3%) of 41 from Zou (Table 3). The difference was not significant.

**Table 3:** Buruli ulcer patient follow-up in villages of the Atlantique and Zou regions, Benin*

Patients treated by surgery at CSNG†	Region		
	Atlantique, n (%)	Zou, n (%)	Total, n (%)
Cured	24 (96.0)	38 (92.7)	62 (93.9)
Recurred	1 (4.0)	3 (7.3)	4 (6.1)
Total	25	41	66

*Pearson p value for the table nonsignificant. †CSNG, Centre Sanitaire et Nutritionnel Gbemoten.

The location, type, and size of previous and new lesions are indicated in Table 4. Three patients had a cutaneous lesion at the previous site, and 1 had a large edematous lesion at a new site (right leg) with bone involvement (right tibia). The follow-up times of these 4 recurrent cases ranged from 12 to 30 months. At CSNG, 57 patients (3.4%, 95% CI 2.6–4.4) of the 1,687 admitted from 1997 to 2001 returned spontaneously to the center with a recurrent lesion (2). The follow-up times of these recurrent cases ranged from 1 to 68 months. Of the 4 recurrent cases, 2 developed within a year, decreasing the recurrence rate within 1 year to 3.0% (95% CI 0.5–11.5). The 2 other recurrent lesions had developed by follow-up visits at 17 and 30 months.

**Table 4:** Location, type, and size of previous and new lesions in 4 Buruli ulcer recurrent cases

Patient no.	Size, type, and location of previous lesion*	Type, size, and location of new lesion*	Time since discharge from hospital (mo)
97-294	Small ulcer, right foot	Small ulcer, same site	30
99-280	Large plaque + edema, left leg	Large plaque + edema, same site	12
–	Small ulcer, left foot	Small ulcer, same site	12
99-227	Large edema, left leg + bone lesion, left tibia	Large edema right leg + bone lesion right tibia	17

*Small, <5 cm diameter; large, >5 cm diameter.

Of the 66 patients treated at CSNG and followed-up in the villages, 10 had received antimycobacterial drugs (streptomycin and rifampin) 1 or 2 days before surgical excision and a few days after surgery; 2 of them had recurrent cases.

## Discussion

The importance of follow-up of Buruli ulcer patients is accepted but little studied. Recurrences of Buruli ulcer are not exceptional (10). Early follow-up is important to rapidly detect recurrent cases and refer patients to treatment. Delays in seeking medical advice can lead to severe complications, including dissemination of disease, especially the development of bone lesions (2,11).

Scheduled programs for repeated follow-up visits of all treated patients would be ideal but are rarely successful in most disease-endemic areas. In our study, only 66 (9.3%) of 707 patients from Ouinhi, Zogbodomey, and Zè treated at CSNG could be followed up. Not all Buruli ulcer–endemic villages could be visited, for financial and logistic reasons. In the 24 visited villages, 44% of former patients were located. If patients were not at home each time we visited them, friends or relatives were able locate them. The patients we could not find were those who lived outside the villages in dwellings that were distant and difficult to reach.

Buruli ulcer is well known to the villagers in disease-endemic areas. In Atlantique and Zou, the field officer, a resident villager, listed all Buruli ulcer patients in his village and guided the team to each patient's house. These guides (often teachers) offered their help for this spontaneously, without compensation, and were motivated by their concern for this health problem. All patients treated at CSNG (recurrent case or not) welcomed the survey team with enthusiasm. However, we cannot exclude the possibility that some patients were not located, especially patients who had been treated by traditional practitioners.

More patients were located in Atlantique (67.7%) than in Zou (36.3%). This finding may be partially due to the fact that most cases in patients from Atlantique were diagnosed from 1999 to 2001, while patients from Zou were treated before 1989. Therefore, locating recent patients from Atlantique was easier than finding those from Zou who were treated >10 years ago. In addition, all patients from Atlantique came from 1 district and were concentrated in geographically restricted areas that were easy to survey. Patients from Zou were more geographically dispersed. Access to some villages and to some houses outside the villages was in general more difficult in Zou districts than in the Atlantique district.

The Buruli ulcer recurrence rate in CSNG-treated patients was low (6.1%) in comparison with those usually reported. Lunn found recurrence rates from <20% to >50% (12). According to WHO, these rates vary from 16% for patients whose conditions are diagnosed early to 28% for patients who seek treatment late (13). In the 2 field studies in Ghana, recurrence rates were 16%–47% (5,6). Even though some rates reported from Ghana are somewhat higher than our highest rate, we do not consider the differences meaningful.

Recurrence rates are directly proportional to the length of follow-up. Muelder and Nourou followed up 28 patients from Sagon (Benin) for up to 42 months and found that the longer the follow-up periods, the higher the accumulative recurrence rate (14). In our study with a follow-up period of up to 7 years, most patients treated at CSNG were in good health.

Two of our recurrent cases did not fit into the definition of recurrences established by WHO, namely, that the recurrence should appear within 1 year of completing treatment (1). In a previous study (11), we noted that some patients came to CSNG with a recurrence >1 year after discharge from the center, most of them with bone lesions. Among the 4 patients who had a recurrence of the disease, a new lesion developed in l patient at a site distant from the initial lesion. This patient had a bone lesion at the time of his first disease episode. Patients with bone lesions are prone to have disseminated lesions at multiple sites (2,11) and to have recurrences >1 year after discharge from the hospital (11). Detecting such disseminated lesions should also imply a follow-up period longer than 1 year. According to our results and those of previous studies, the WHO definition of recurrence should be revised to include a follow-up period longer than 1 year ("delayed recurrence").

As for other disease such as tuberculosis (15) or urinary tract infections (16), distinguishing recurrence or relapse (endogenous recurrent infection) from reinfection (exogenous recurrent infection) is important in Buruli ulcer. This distinction cannot yet be rigorously made for Buruli ulcer, but considering a second lesion that appears next to or within the first lesion as relapse seems reasonable. In the case of bone lesions, however, we propose the term relapse can also be applied even if the second lesion is situated far from the first lesion (11). Recurrence at a different site may result from hematogenous or lymphatic spread of the etiologic agent from earlier *M. ulcerans* disease at a different site. In this case, bone is almost always associated with new lesions; however, they may result from reinfections. The development of fingerprinting molecular biology tools, for example, restriction fragment length polymorphism (17) and mycobacterial interspersed repetitive unit–variable-number tandem repeat (18), seems promising for the resolution of this problem.

Reinfection seems to be infrequent. Gooding et al. demonstrated that when Buruli ulcer does not develop in persons who have been exposed to *M. ulcerans*, they have probably developed an immune response to *M. ulcerans* (19). This finding confirms the earlier hypothesis that disease develops in only a portion of exposed persons in Buruli ulcer–endemic areas (20). In addition, small, self-healing minor ulcers may go undetected or dismissed by the patient (21).

Treatments other than surgery also led to recurrences. Meyers et al. (22) reported 2 recurrent cases among 6 Buruli ulcer patients after heat treatment. The follow-up periods of the 6 patients ranged from 3 to 22 months. In both patients, the recurrence was at a site distant from the heat-treated lesion, and no evidence showed that the initial lesion had reactivated or that the new lesions represented extension of the heat-treated ulcers. In fact, 1 of the 2 patients had 2 previous recurrences, and the other returned 19 months after hospital discharge with new, large Buruli lesions on ankles and lower legs. The authors suggested a probable reinfection from the environment.

Some antimycobaterial drugs are effective against *M. ulcerans* in vitro (23,24) and in vivo in animals (25,26), but the effect of antibacterial treatment in humans remains obscure (27,28). Recently, Grosset (4) demonstrated that early forms of Buruli ulcer may be treated by a combination of streptomycin and rifampicin. In our study, the number of patients who received antimycobacterial treatment was small (10), and the duration of treatment was too short (1–2 weeks) to expect any noticeable effect. Moreover, 2 of our recurrent case-patients had received antimycobacterial treatment. Therefore antimycobacterial use would not be expected to explain, even in part, the low rates of recurrence observed in the present study. Antibiotherapy is considered an adjuvant or complementary treatment to surgery. Effective bactericidal drugs for humans remain a research priority and may play a role in reducing recurrences.

Few health professionals are knowledgeable about Buruli ulcer or have worked in Buruli ulcer–endemic areas. Buruli ulcer does not yet appear in health statistics, and few physicians or surgeons are trained to treat this disease. Recurrences of Buruli ulcer could likely be reduced by improving training of doctors in correct excision procedures (6) and by following up patients regularly.

Not all villages of the Buruli ulcer–endemic districts chosen in the present study were known to be Buruli ulcer–endemic. As shown in Table 1, Buruli ulcer was endemic in 70 of 173 villages (40.5%). Although not the object of the present study, Buruli ulcer–nonendemic villages should be studied further to determine their true level of Buruli ulcer endemicity. Further research should also compare Buruli ulcer–endemic and Buruli ulcer–nonendemic villages from the same district to determine which factors may play a role in the prevalence of Buruli ulcer (29).

In conclusion, our study showed that the recurrence rate after surgery at CSNG after a follow-up period ranging from 2 months to 7 years is low. Creating regional centers that allow patients easy access to treatment with short travel distances and low treatment costs, coupled with educational sessions, could help other centers attract and treat most Buruli ulcer patients in their region. This proximity would render the follow-up of patients easier and be a source of new information on the disease for the population. This process would lessen the stigma of Buruli ulcer by considering it a disease and limiting the number of Buruli ulcer patients who attend traditional healers. Research to develop an effective antimycobacterial treatment remains a priority, and progress in this area may alleviate the problem of recurrences. New molecular tools may help differentiate recurrence and reinfection and clarify the definition of a recurrent case.
